# Nonsteroidal anti-inflammatory drug use and Alzheimer's disease risk: the MIRAGE Study

**DOI:** 10.1186/1471-2318-5-2

**Published:** 2005-01-12

**Authors:** Agustín G Yip, Robert C Green, Matthew Huyck, L Adrienne Cupples, Lindsay A Farrer, for the MIRAGE Study Group

**Affiliations:** 1 Department of Medicine (Genetics Program), Boston University School of Medicine, 715 Albany Street, Boston MA 02118-2526, USA; 2 Department of Neurology, Boston University School of Medicine, 715 Albany Street, Boston MA 02118-2526, USA; 3 Department of Genetics and Genomics, Boston University School of Medicine, 715 Albany Street, Boston MA 02118-2526, USA; 4 Department of Epidemiology, Boston University School of Public Health, 715 Albany Street, Boston MA 02118-2526, USA; 5 Department of Biostatistics, Boston University School of Public Health, 715 Albany Street, Boston MA 02118-2526, USA

## Abstract

**Background:**

Nonsteroidal anti-inflammatory drugs (NSAID) use may protect against Alzheimer's disease (AD) risk. We sought examine the association between NSAID use and risk of AD, and potential effect modification by APOE-ε4 carrier status and ethnicity.

**Methods:**

The MIRAGE Study is a multi-center family study of genetic and environmental risk factors for AD. Subjects comprised 691 AD patients (probands) and 973 family members enrolled at 15 research centers between 1996 and 2002. The primary independent and dependent variables were prior NSAID use and AD case status, respectively. We stratified the dataset in order to evaluate whether the association between NSAID use and AD was similar in APOE-ε4 carriers and non-carriers. Ethnicity was similarly examined as an effect modifier.

**Results:**

NSAID use was less frequent in cases compared to controls in the overall sample (adjusted OR = 0.64; 95% CI = 0.38–1.05). The benefit of NSAID use appeared more pronounced among APOE-ε4 carriers (adjusted OR = 0.49; 95% CI = 0.24–0.98) compared to non-carriers, although this association was not statistically significant. The pattern of association was similar in Caucasian and African Americans.

**Conclusions:**

NSAID use is inversely associated with AD and may be modified by APOE genotype. Prospective studies and clinical trials of sufficient power to detect effect modification by APOE-ε4 carrier status are needed.

## Background

Several cross-sectional [1,2], case-control [3-6], and prospective studies [7-9] have reported an inverse association between nonsteroidal anti-inflammatory drug (NSAID) use and the risk of Alzheimer's disease (AD), whereas others [10-12] have not. In this report, we present results of analyses of data from the Multi-Institutional Research in Alzheimer's Genetic Epidemiology (MIRAGE) Study in which we examined potential effect modification by APOE-ε4 carrier status and ethnicity on this association.

## Methods

### Subjects and data collection

The MIRAGE Study is a multi-center family study of genetic and environmental risk factors for AD sponsored by the National Institute on Aging since 1991. The details of MIRAGE Study data collection procedures, protocols for obtaining family histories, and reports of validity studies of the MIRAGE questionnaires have been published elsewhere. [13-15] Briefly, families were recruited through probands meeting NINCDS-ADRDA criteria [16] for probable or definite AD who were ascertained through research registries and memory clinics. After obtaining informed consent from non-demented family members, and a combination of consent or assent – along with informed consent by proxy – on living demented subjects, questions eliciting demographic data and information about presumptive risk factors for AD were obtained using standardized MIRAGE questionnaires.

Questions pertaining to NSAID use were added to the questionnaire in 1996, and the data presented in this report were collected from May, 1996 through May, 2002. Questions about the proband were answered by a surrogate source within the family, typically the spouse or adult offspring. The same information was sought on non-demented first-degree family members of these probands over 50 years of age, usually a sibling or spouse (less commonly parents or children).

1020 family members in this analysis claimed to be cognitively normal, or were reported by family informants to be dementia-free. Of these, 982 were evaluated using the modified Telephone Interview of Cognitive Status (mTICS) [17,18], and normal cognitive status was confirmed in 973 (99.1%). Information on both patients and first-degree family members was supplemented where available by multiple informants, and medical and nursing home records.

To elicit information on prior NSAID use, the following question was asked: "Have you ever taken a nonsteroidal anti-inflammatory medication (e.g. Advil, Motrin, etc.) *on a daily basis for more than 6 months*?" No distinction was made between aspirin and other classes of NSAIDs. For proxy reporting about a relative with AD, the question substituted "your relative" for "you". For any affirmative answer, a follow-up question asked for the dates at which the medications were first used and the names of all NSAIDs that had been used.

A discrete "index date" was established within each family corresponding to the earliest date that the family or medical records reported AD symptoms to have begun in the proband. Subjects from each family (whether AD cases or non-demented family members) were considered to have been exposed to NSAIDs only if the starting date for NSAID use preceded this index date by at least one year. Age represented the age of cases and of non-demented relatives at the index date, and was treated as a continuous variable.

**Figure 1:**
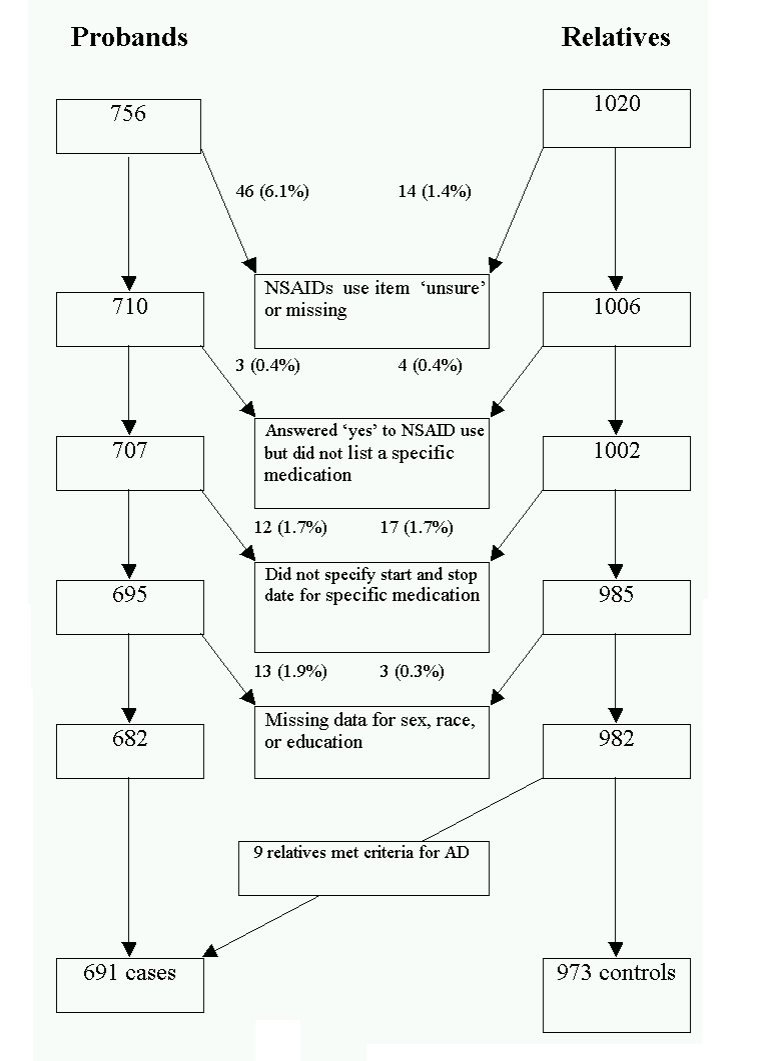
MIRAGE subjects ≥ 50 years (adjusted for family index date) with APOE genotype who completed the personal history questionnaire.

As shown in Figure 1, there were 756 probands and 1020 relatives over the age of 50 with APOE genotype who were queried about prior NSAID use. After exclusions for those subjects who had missing or unsure responses for the name of their medication, did not include a medication start date, or had missing data for the variables age, sex, education or ethnicity, there remained for analysis 682 probands and 982 relatives. Of the 982 relatives, nine were reported to be demented with the onset of their dementia prior to the index date for that family, and their diagnoses were verified by review of medical records as having probable or definite AD by research criteria, so these were classified with the probands as having AD.

### Statistical analysis

Analyses were performed using SAS version 8.2. The primary independent and dependent variables were prior NSAID use and AD case status, respectively. Crude odds ratios were computed in the first instance, followed by adjusted estimates using generalized estimating equation (GEE) models [19] to account for the possibility that variables of interest (e.g., medication use, APOE status) could be correlated among individuals within families. Adjustments were made for the following covariates: age, sex, ethnicity (categorized as White, African-American, or other), education (less than versus equal to or greater than high school level), and APOE-ε4 carrier status (one or two ε4 alleles vs. none).

We stratified the dataset in order to evaluate whether the association between NSAID use and AD was similar in APOE-ε4 carriers and non-carriers. In addition, we formally evaluated these associations by adding an interaction term (ε4 * NSAID use) to the GEE model. Ethnicity was similarly examined as an effect modifier.

## Results

Characteristics of the 1664 subjects are listed in Table 1. AD patients were more likely to be older and to be APOE-ε4 carriers compared to controls. The distributions of sex and education were not different between cases and controls.

**Table 1:** Characteristics of AD patients and non-demented family

CHARACTERISTIC	AD (N = 691)	NON DEMENTED (N = 973)	AGE ADJUSTED PERCENT AD	AGE ADJUSTED PERCENT NON-DEMENTED	P-VALUE*
Mean Age (SD)	70.0 (8.2)	65.0 (8.8)			<0.0001
Sex (%male)	242 (35.0)	381 (39.2)	36.5	39.9	0.39
Greater than HS Ed (%)	403 (58.3)	643 (66.1)	59.3	65.1	0.10
African American (%)	215 (31.1)	204 (21.0)	28.4	21.3	0.01
Use of NSAIDs† (%)	24 (3.5)	66 (6.8)	3.5	6.7	0.08
APOE-ε4 carrier (%)	448 (64.8)	370 (38.0)	65.3	38.0	<0.0001

* Reported p-values use General Estimating Equations to account for correlation among observations.

†Exposure to non-steroidal anti-inflammatory drugs had to precede index date by one year or more.

Sixty-six out of 973 non-demented relatives (6.8%) and 24 of 691 cases (3.5%) reported previous NSAID use (odds ratio = 0.49; 95% CI = 0.31–0.80). After adjustment for age, sex, educational level, and ethnicity, the odds ratio (OR) of NSAID use among AD cases compared to non-users was 0.57 (95% CI = 0.35–0.93); it was 0.64 (95% CI = 0.38–1.05) when APOE carrier status was added to the GEE model (see Table 2).

**Table 2:** Risk of AD with and without prior use of NSAIDs, stratified by APOE-ε4 carrier status

EXPOSURE	AD (N = 691)	NON-DEMENTED FAMILY MEMBERS (N = 973)	CRUDE ODDS RATIO (95% CI)	AGE-ADJUSTED ODDS RATIO (95% CI)	ADJUSTED ODDS RATIO (95% CI)
**Overall**					
No NSAIDs	667	907	1.0	1.0	1.0
Use of NSAIDs	24	66	0.49 (0.31, 0.80)	0.55 (0.34, 0.88)	0.64 (0.38, 1.05)*
**Having no ε4 alleles**					
No NSAIDs	231	556	1.0	1.0	1.0
Use of NSAIDs	12	47	0.61 (0.32, 1.18)	0.75 (0.38, 1.46)	0.78 (0.39, 1.52)**
**Having at least one ε4 allele**					
No NSAIDs	436	351	1.0	1.0	1.0
Use of NSAIDs	12	19	0.51 (0.24, 1.06)	0.47 (0.24, 0.96)	0.49 (0.24, 0.98)**

*Adjusted for age, sex, ethnicity, education, and APOE-ε4 status.

**Adjusted for age, sex, ethnicity, and education.

***NSAID*APOE-ε4 interaction p-value = 0.04.

The magnitude of inverse association was greater among APOE ε4 carriers (OR = 0.49; 95% CI = 0.24–0.98) than non-carriers (OR = 0.78; 95% CI = 0.39–1.52). However, formal evaluation of the interaction between NSAID use and APOE-ε4 carrier status did not reveal a significant difference (p = 0.40). The association between NSAID use and AD risk was similar among Caucasian and African Americans (data not shown).

## Discussion

This study supports the findings of previous reports [1-6] suggesting that use of NSAIDs for at least six months is associated with a reduced risk of AD. This association appears to be more robust among APOE-ε4 carriers than non-carriers, although the difference in associations between these two groups was not statistically significant.

The MIRAGE Study includes the largest number of well-characterized AD cases and family controls to date, and this large sample size permits adjustment for important potential confounders, as well as the power to examine effect modification by APOE genotype and ethnicity. The subjects without dementia were first-degree family members of AD cases, providing some degree of informal matching on age, socioeconomic status, and health-seeking behavior.

However, these results must be interpreted in light of some methodological limitations. Data on NSAID use was collected with a single retrospective question that did not distinguish between aspirin use and non-aspirin NSAID use. Moreover, while non-demented participants reported on themselves, a proxy historian reported on most of the demented individuals.

Differential reporting is a potential source of bias in a study that uses self-report on most of the non-demented subjects, yet relies on surrogate respondents for all of the subjects with AD. Asymmetric data collection is difficult to avoid when cases are cognitively impaired, but may be more accurate than expected in AD patients where the surrogate historian has a long association with the subject. We addressed this potential bias by performing an independent validation study to determine the accuracy of surrogate information on a number of questions, including the same questions used in this report about NSAID use. [15] This study found substantial reliability on the NSAID item (kappa = 0.70). While a validation study comparing proxy historians for non-demented persons does not perfectly mirror the situation in which proxy historians report on demented individuals, our study revealed excellent concordance for surrogate responses from most categories of relatives.

This result is consistent with those of prior studies which found a similar association despite differences in study design (cross-sectional [1,2] vs. case-control [3-6,10,11] vs. prospective [7-9,12]), sampling frame (family members [10] vs. registry-based [11] vs. general population [1-9,12]), ascertainment of exposure, type of medication considered (aspirin [2-4,6,8-10,12] vs. non-aspirin NSAIDs [1-11] vs. 'any' NSAID [4,8]), duration of exposure (current [1-3] vs. any history of use, duration ranging from a week to at least six months [3,5-12]), and degree of matching or adjustment (usually adjusted for age, sex, and education, less frequently APOE genotype [7-9,12]).

While many studies have examined the association between NSAID use and risk of AD, few have examined the impact of APOE genotype on this association. The Cache County Study [9,20], the Canadian Study for Health and Aging (CSHA) [8], and the Rotterdam Study [7] adjusted for APOE and tested for effect modification and found none. But they had fewer AD cases, and, in the CSHA had a smaller proportion of genotyped subjects. The Rotterdam Study [7] reported separate odds ratios for APOE-ε4 carriers and non-carriers, but this sample did not have any subjects who were both APOE-ε4 carriers and who reported long-term use of NSAIDs. They found no difference in risk between those with at least one ε4 allele compared to ε4 non-carriers among subjects who used NSAIDs between one month and two years.

Our data suggest an enhanced protective benefit of NSAID use among those with ε4. A smaller protective effect was also evident among those lacking ε4. In our sample there were relatively few AD cases who were not ε4 carriers, thus the appearance of different patterns of association between NSAID use and AD risk among APOE genotype subgroups may be spurious. This difference could also have arisen as a result of bias and confounding. The genotype-specific association could be explained by differential inclusion of subjects into the study on the basis of APOE-ε4 carrier status and NSAID use. This might occur if there were differential mortality, according to APOE genotype, among those with AD who had a history of NSAID use; or if for any reason among NSAID users APOE-ε4 carriers were less likely than non-carriers to be diagnosed with AD (or conversely, if among non-users of NSAIDs AD was more likely diagnosed in APOE-ε4 carriers compared to non-carriers). Differential recall could also give rise to this observation. However, these explanations are unlikely because subjects were not selected on the basis of APOE genotype. It is also possible that APOE-ε4 carrier status is a proxy for differentially distributed unmeasured confounders related to NSAID use such as inflammatory disease processes.

Alternatively, our results imply that NSAID use affects AD risk differently between APOE-ε4 carriers and non-carriers. For example, because ε4 carriers are inherently more vulnerable to AD, there is a greater opportunity for attributable risk reduction. This explanation does not imply biological interaction between NSAIDs and ε4. On the other hand, the ε4 isoform may have greater pro-inflammatory properties [21] and ε4 individuals may be more responsive to the benefits of NSAID use than those lacking ε4.

Examination of this finding in prospective studies and clinical trials of sufficient power (such as the ADAPT Study [22], a prospective trial of anti-inflammatory use in the prevention of AD) to detect effect modification by APOE-ε4 carrier status is needed. Such confirmation would provide critical insights into the mechanisms by which APOE isoforms modulate AD risk and into novel therapeutic strategies.

## Conclusions

NSAID use is inversely associated with AD and may be modified by APOE genotype. Prospective studies and clinical trials of sufficient power to detect effect modification by APOE-ε4 carrier status are needed.

## Competing interests

The author(s) declare that they have no competing interests.

## Authors' contributions

Study concept and design (LAF, RCG, LAC); acquisition of data (LAF, RCG, MIRAGE investigators); analysis and interpretation of data (AGY, MH); drafting of the manuscript (AGY, RCG, LAF); critical revision of the manuscript for important intellectual content (AGY, LAF, RCG, LAC); statistical expertise (LAC); obtained funding (LAF, RCG, LAC, MIRAGE investigators). All authors read and approved the final manuscript.

## Pre-publication history

The pre-publication history for this paper can be accessed here:


